# Summer dreams

**DOI:** 10.3201/eid2310.171117

**Published:** 2017-10

**Authors:** Veranja Liyanapathirana

**Affiliations:** Author affiliation: University of Peradeniya, Peradeniya, Sri Lanka

**Keywords:** Dengue, mosquitoes, *Aedes*, *aegypti*, *albopictus*, rain, food, rambutan, rurian, peels, vector

Monsoon rains

Have come and gone

Quintessential

Remnants of summer

Rambutans and Durians

Discarded peels

Bustles with activity

Lives of other kinds

Wriggling and swimming

Summer dreams

Of warm humid nights

Fanning away the creatures of nature

That annoying bustle of wings

Of lives that emerged from the discards

Creatures of nature

That fly in search of meals

Meals of red

Within the meal

Enveloped

In a protective sheath

Lies a being

Is it alive or is it not?

We argue in the summer’s heat

How to define life?

This being is a villain

A villain of a kind

Minute yet mighty

Astute and elusive

Adapting and evolving

